# Cluster imaging of multi-brain networks (CIMBN): a general framework for hyperscanning and modeling a group of interacting brains

**DOI:** 10.3389/fnins.2015.00267

**Published:** 2015-07-28

**Authors:** Lian Duan, Rui-Na Dai, Xiang Xiao, Pei-Pei Sun, Zheng Li, Chao-Zhe Zhu

**Affiliations:** ^1^State Key Laboratory of Cognitive Neuroscience and Learning, Beijing Normal UniversityBeijing, China; ^2^IDG/McGovern Institute for Brain Research, Beijing Normal UniversityBeijing, China; ^3^Center for Collaboration and Innovation in Brain and Learning Sciences, Beijing Normal UniversityBeijing, China

**Keywords:** social interaction, graph theory, functional near-infrared spectroscopy, hyperscanning, social neuroscience, multi-brain network

## Abstract

Studying the neural basis of human social interactions is a key topic in the field of social neuroscience. Brain imaging studies in this field usually focus on the neural correlates of the social interactions between two participants. However, as the participant number further increases, even by a small amount, great difficulties raise. One challenge is how to concurrently scan all the interacting brains with high ecological validity, especially for a large number of participants. The other challenge is how to effectively model the complex group interaction behaviors emerging from the intricate neural information exchange among a group of socially organized people. Confronting these challenges, we propose a new approach called “Cluster Imaging of Multi-brain Networks” (CIMBN). CIMBN consists of two parts. The first part is a cluster imaging technique with high ecological validity based on multiple functional near-infrared spectroscopy (fNIRS) systems. Using this technique, we can easily extend the simultaneous imaging capacity of social neuroscience studies up to dozens of participants. The second part of CIMBN is a multi-brain network (MBN) modeling method based on graph theory. By taking each brain as a network node and the relationship between any two brains as a network edge, one can construct a network model for a group of interacting brains. The emergent group social behaviors can then be studied using the network's properties, such as its topological structure and information exchange efficiency. Although there is still much work to do, as a general framework for hyperscanning and modeling a group of interacting brains, CIMBN can provide new insights into the neural correlates of group social interactions, and advance social neuroscience and social psychology.

## Introduction

Exploring the neural mechanisms of human social interactions is the core of social neuroscience. In the past decade, with the help of “hyperscanning” techniques (Montague et al., 2002; Babiloni et al., 2006; Funane et al., 2011; Baess et al., 2012; Cui et al., 2012; Hirata et al., 2014), many studies have investigated the neural correlates of two-person social interactions (see Hari and Kujala, 2009; Astolfi et al., 2011; Sänger et al., 2011; Konvalinka and Roepstorff, 2012; Liu and Pelowski, 2014 for reviews). However, studying the neural correlates of more than two people's social interactions remains quite a new area. Novel neuroimaging technology for “crowd psychology” studies (Hari and Kujala, 2009) is still awaiting further development.

In recent years, some pioneering studies have explored using functional magnetic resonance imaging (fMRI) (Tomlin et al., 2013; Smith et al., 2014) and electroencephalogram (EEG) (Babiloni et al., 2006, 2007b, 2011, 2012) to simultaneously scan multiple interacting brains (3–5 participants), taking large steps from two-brain neuroscience to multi-brain neuroscience. However, some technical challenges still limit the further development of this new area. First, from the aspect of brain imaging technology, fMRI-based multi-person hyperscanning usually demands one fMRI scanner for each participant, making it difficult to scan larger groups (although recently a novel technique allowed a single fMRI scanner accommodating two participants to scan them at the same time, Lee et al., 2012). Moreover, the confines of an fMRI scanner are unlike daily-life environments for social interaction. Compared with fMRI, EEG allows more naturalistic scanning environments and has a cost advantage which allows the integration of more EEG recording systems to scan more participants. However, EEG cannot precisely localize neuroelectrical signal origins (Michel et al., 2004), which increases the difficulty in understanding experimental results.

Second, from the aspect of modeling and analysis, a current challenge is how to effectively model the complex interaction process among all interacting brains. Social interaction relies upon participants' information exchange (Tognoli et al., 2007). In a multi-person interaction environment, the information exchange process is much more complex than that in a two-person situation. Therefore, single-brain level analysis and paired-brain level analysis may not support comprehensive study of the characteristics of multi-brain interaction processes.

Confronting these challenges, in the present study we propose a general framework for hyperscanning and modeling multiple interacting brains, called “Cluster Imaging of Multi-brain Networks” (CIMBN). CIMBN consists of two parts. The first part is a simultaneous multi-brain imaging technique based on multiple functional near-infrared spectroscopy (fNIRS) recording systems, called “cluster imaging.” FNIRS is a fast-developing brain imaging technology. It measures the hemodynamic responses of neural activity, and has relatively high spatial resolution (about 1–3 cm, Boas et al., 2004) and capacity for signal localization. FNIRS is quiet, comfortable, and insensitive to participants' body- and eye-movements, allowing participants to communicate with each other verbally and non-verbally just like in daily life (Jiang et al., 2012). Compared with fMRI, fNIRS has much lower cost. Therefore, it is feasible to simultaneously scan more participants by increasing the number of fNIRS instruments. Moreover, most commercially available fNIRS systems provide dozens of measurement channels. Therefore, a single fNIRS instrument can be used to measure multiple participants, at the cost of reduced measurement area for each participant. These unique advantages make fNIRS very promising for developing extensible cluster imaging systems of high ecological validity.

The second part of CIMBN is a multi-brain network (MBN) modeling method based on graph theory and network theory. These approaches give powerful tools for modeling and analyzing the connectivity relationships and information transmission features between brains. Network analysis techniques have been widely used in individual neuroscience to study the brain's structural and functional architecture (Bullmore and Sporns, 2009, 2012; Park and Friston, 2013). Recently it has also been used in two-brain hyperscanning studies (Babiloni et al., 2007a; Fallani et al., 2010; Sänger et al., 2012, 2013). In the framework of CIMBN, a network is used to model the interacting relationships among more than two brains. The idea is to abstract each brain as a network node, and then take some index of brain-to-brain interaction (e.g., the neural synchronization) between each pair of brains as the strength of the network edge between the nodes. Then the information transmission features of this MBN can be characterized by its topological properties. As a new way to analyze a group's hyperscanning data, this approach has the potential to provide new insights into the neural correlates of information exchange among interacting people.

In this technical report, a cluster imaging platform consisting of two fNIRS systems with parallel imaging capacity of nine participants is described. A simple nine-person drumming experiment was conducted to demonstrate the feasibility of the platform. Concepts, methods, and application of MBN modeling are introduced. Finally, as a general framework for social neuroscience and social psychology studies, the advantages, limitations, and possible future improvements of CIMBN are discussed.

## Cluster imaging of multi-brain networks

### Part I: fNIRS-based cluster imaging system

**Figure 1:**
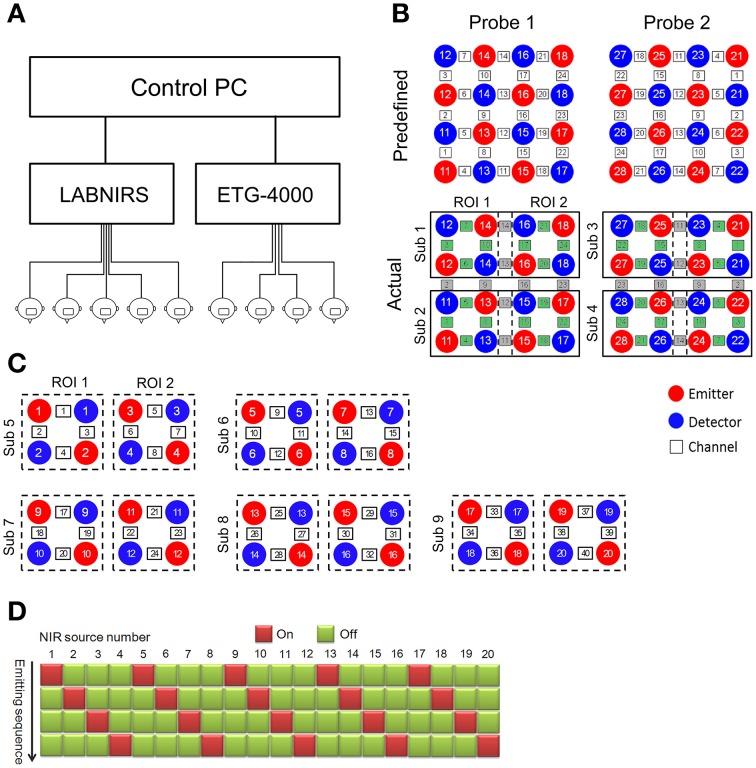
**Architecture and channel configuration schematic of the fNIRS-based cluster imaging system**. **(A)** System architecture diagram. **(B)** Channel configuration of ETG-4000. Upper panel shows the two predefined probes with 4 × 4 configuration. Lower panel shows the actual channel allocation used in multi-brain imaging. Green channels are used while gray channels are discarded. **(C)** Channel configuration of LABNIRS. **(D)** Activation sequence diagram of the LABNIRS light sources.

In this study, the cluster imaging system consisted of two fNIRS systems (one ETG-4000, Hitachi Medical Corporation, Japan; one LABNIRS, Shimadzu Corporation, Japan). Both fNIRS systems were connected to a personal computer (Intel i3 CPU, 4GB RAM, Microsoft Windows XP operating system) to receive control commands. The ETG-4000 was connected via the RS-232 serial port, and the LABNIRS was connected via the IEEE-1284 parallel port. Custom-developed software (programmed using MATLAB, the MathWorks Company) running on the PC was used to send triggers, experimental markers, and timestamps to the two fNIRS systems (Figure 1A).

The optical fibers of each fNIRS system were divided into several bundles to scan multiple participants. Specifically, the ETG-4000 was used to scan four participants and the LABNIRS was used to scan five participants. Each participant was allocated eight near-infrared optodes (four sources and four detectors). When configuring the measurement channels, it should be noted that there are two different ways that multi-channel fNIRS systems operate. One way is frequency-division multiplexing. This type of fNIRS system modulates every near-infrared (NIR) light source at a different frequency, therefore enabling frequency-based demultiplexing of the mixed signal received by each detector into components from different NIR light sources. For this type of fNIRS system (e.g., the ETG-4000), the matching between the source and detector optodes are predetermined. Therefore, experimenters need to divide the measurement channels based on the system-predefined channel configuration. For example, as the ETG-4000 predefined two 4 × 4 probe sets, they were divided into eight subsets to scan four participants in our cluster imaging system [two subsets for each participant, covering two regions of interest (ROI), Figure 1B].

Another way multi-channel fNIRS systems identify signals from different NIR light sources is by time-division multiplexing, in which the light sources are activated sequentially to avoid aliasing. For this type of fNIRS system (e.g., the LABNIRS), the experimenter can customize the source-detector arrangement and set the source activation sequence to realize the scanning of multiple participants simultaneously (Figures 1C,D).

In the current system, the optical fiber lengths of both ETG-4000 and LABNIRS are 3 m, allowing the nine participants to communicate in a face-to-face manner in a wide room. However, limited by the arrangement of the optical fibers, the participants cannot ambulate freely in the room. To study social interactions among a group of ambulating people (e.g., similar to a cocktail party), wireless fNIRS systems should be used.

### Part II: Multi-brain network (MBN) modeling and analysis

Network theory provides promising tools for analyzing the complex information exchange process among multiple interacting brains. To conduct MBN analysis, the first step is to define the nodes and edges. As our fNIRS multi-brain imaging setup only covers a few ROIs, instead of the whole brain, we define a node as one ROI in one brain. The choice of ROI is determined according to the experimental hypothesis. In our case, to investigate social functions, the prefrontal cortex or temporal parietal junction were chosen. After determining the nodes, one of several inter-brain synchronization measures can be used to assign the weights of the network edges, such as the correlation coefficient (Holper et al., 2013), coherence (Cui et al., 2012), and mutual information (Naeem et al., 2012). Alternatively, considering the direction of information transfer, the edge weights can be calculated by using dynamic causal modeling (Friston et al., 2003) or Granger-causality (Holper et al., 2012). Edge weights are stored in a connection matrix where the element in the *i*-th row and *j*-th column is the inter-brain neural synchronization between nodes *i* and *j*. An optional step is to threshold the connection matrix to generate a binary adjacency matrix, forming an unweighted network. Finally, the connection matrix can be visualized as a graph to intuitively show the structure of the network.

After the MBN is built, a variety of topological properties of the MBN can be calculated to quantitatively assess the network's information exchange characteristics. There are two main categories of topological measures. One category consists of nodal metrics which describe the topological properties of nodes, such as nodal degree, nodal clustering coefficient, betweenness, and nodal efficiency. The other category consists of network metrics which describe the topological properties of the network, such as clustering coefficient, characteristic path length, network efficiency (global efficiency and local efficiency), assortativity, hierarchy (Ravasz and Barabasi, 2003), synchronization (Barahona and Pecora, 2002), modularity (Girvan and Newman, 2002), as well as the small-world property (Watts and Strogatz, 1998) and scale-free property (Barabasi and Albert, 1999). These metrics of the MBN can be correlated with indices of social interaction behaviors to investigate the neural correlates of multi-person social interactions. For example, a group member with high nodal degree (many direct connections with other nodes) or high betweenness (as an important mediating node by which other nodes are connected) in the MBN may show high leadership in the group, because a leader usually needs to exchange information with other members more frequently, which may make his/her brain an important “hub” in the MBN. As another example, the task performance of a group of people may be correlated with the MBN's efficiency indices, because higher information exchange efficiency among brains may lead to better communication, comprehension, and cooperation.

## A demonstration experiment

### Cluster imaging of a nine-person drumming experiment

**Figure 2:**
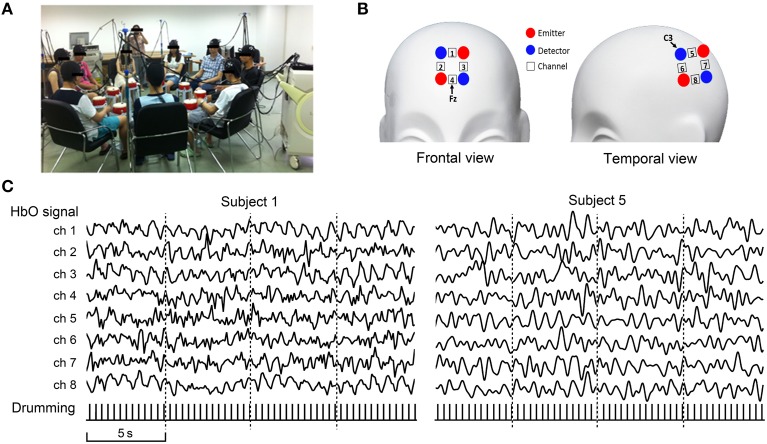
**Nine-person drumming recorded using the fNIRS-based cluster imaging system**. **(A)** Photo of simultaneous nine-person cluster imaging during drumming interaction. **(B)** Brain regions (ROI) measured for each participant. **(C)** Signal samples from two representative participants. Recorded oxygenated hemoglobin (HbO) signals were off-line preprocessed, including alignment between the two fNIRS systems using timestamps, down-sampling of LABNIRS data (55.5 Hz) to the sampling rate of ETG-4000 data (10 Hz), and low-pass filtering (2.5 Hz) to remove high-frequency instrumental noise. Bottom row shows behavioral data (drum beats).

As a demonstration of the CIMBN method, we conducted a simple experiment in which nine participants drummed together while their brain activities were simultaneously recorded and analyzed off-line. As shown in Figure 2A, nine participants sat in a circle (about 1.5 m in diameter) in a wide room, each with a drum. They drummed together and tried their best to make their beats consistent with each other, a traditional social interaction task which closely mimics some situations in daily life (Cohen et al., 2014). During the drumming, the brain activities from two regions (the prefrontal cortex and the left temporal parietal junction, Figure 2B) of all nine participants were simultaneously recorded. The signals were then off-line preprocessed, including a temporal alignment between the two fNIRS instruments using the timestamps, a down-sampling of the LABNIRS data (55.5 Hz) to the sampling rate of the ETG-4000 data (10 Hz), and a low-pass filter (2.5 Hz) to remove high-frequency instrumental noise. To obtain the drumming behavioral data, the drum beats of all participants were recorded using vibration transducers and synchronized with the NIRS signals using timestamps.

### Multi-brain network analyses

As an example of MBN construction, we defined the nodes as the prefrontal cortex ROI. For each participant, the fNIRS oxygenated hemoglobin (HbO) signals from the four channels in the prefrontal cortex were ROI-averaged. Edge weights were defined as the Pearson correlation coefficients between the ROI-averaged time courses of each pair of participants. A 9 × 9 connection matrix was obtained by calculating the correlation coefficients for all node pairs. The connection matrix was thresholded into an unweighted binary network with an exploratory threshold of 0.5 and then visualized as a graph. After the MBN construction, some basic indices—degree, nodal global efficiency, nodal local efficiency, network global efficiency, network local efficiency, and nodal betweenness—were calculated. The degree of a node is defined as the number of edges linking to that node. The nodal global efficiency of a node *i* is defined as

(1) $$E_{i\_glob}=\frac{1}{N-1}\sum_{i\neq j\in G}\frac{1}{l_{ij}},$$

where *G* is the graph. *N* is the number of nodes in *G. l_ij_* is the shortest path length between nodes *i* and *j*. The nodal local efficiency of a node *i* is defined as

(2) $$E_{i\_loc}=\frac{1}{N_{G_{i}}(N_{G_{i}}-1)}\sum_{i,j\in G_{i}}\frac{1}{l_{ij}},$$

where *N_G_i__* is the number of nodes in the subgraph *G_i_*, the set of neighboring nodes of node *i*. The network global efficiency is defined as

(3) $$E_{glob}=\frac{1}{N\left(N-1\right)}\sum_{j\neq i\in G}\frac{1}{l_{ij}}.$$

The network local efficiency is defined as

(4) $$E_{loc}=\frac{1}{N}\sum_{i\in G}E_{glob}\left(G_{i}\right).$$

The nodal betweenness of a node *i* describes the number of shortest paths between pairs of other nodes which pass through node *i*. It is defined as

(5) $$B_{i}=\sum_{j\neq i\neq k\in G}\frac{\sigma_{jk}\left(i\right)}{\sigma_{jk}}\;.$$

where σ*_jk_* is the number of shortest paths from node *j* to node *k*, and σ*_jk_*(*i*) is the number of shortest paths from node *j* to node *k* that pass through node *i*.

### Results

Figure 2C shows the oxygenated hemoglobin (HbO) signals from all eight channels of two sample participants (Left: Participant 1, collected using ETG-4000; Right: Participant 5, collected using LABNIRS). The pulse signals below the HbO traces illustrate the drumming behavioral data.

**Figure 3:**
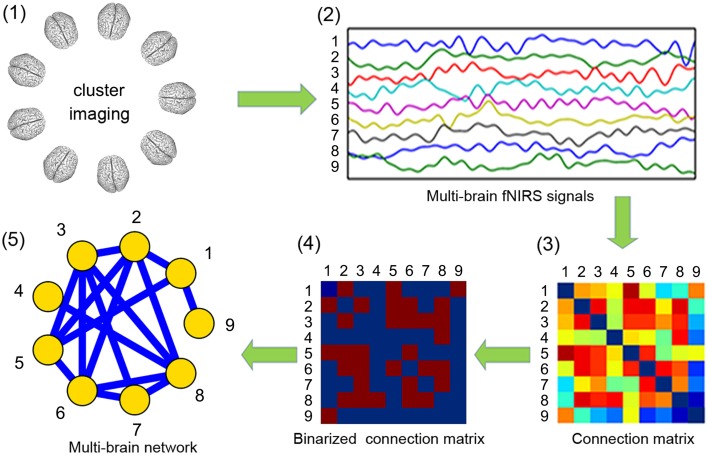
**Flowchart of the construction of a multi-brain network from the nine-person drumming interaction**. All nine participants' brain activities were simultaneously recorded using our fNIRS cluster imaging system (step 1), and the oxygenated hemoglobin (HbO) signals from the four channels in the prefrontal cortex were ROI-averaged (step 2). Edge weights were obtained by calculating the Pearson correlation coefficients between the ROI-averaged time courses, forming a 9 × 9 connection matrix (step 3). The connection matrix was thresholded into an unweighted binary network (step 4) and then visualized as a graph (step 5).

Figure 3 shows the flowchart and the result of each step in constructing the MBN. As illustrated, each pair of participants were either linked directly or indirectly through other nodes, which means all nodes (drummers) are in one connected component. Table 1 shows several basic indices of the MBN: the nodal degree, the nodal efficiency (global and local), the network efficiency (global and local), and betweenness.

**Table 1:** **Indices of the multi-brain network**.

**Participant**	**Nodal**									**Network**
	**1**	**2**	**3**	**4**	**5**	**6**	**7**	**8**	**9**	
Degree	3	5	5	1	4	5	3	5	1	
Global efficiency	0.65	0.81	0.79	0.49	0.73	0.79	0.64	0.79	0.44	0.68
Local efficiency	0.33	0.78	0.85	0	0.83	0.85	1	0.55	0	0.58
Betweenness	7	7.9	2.3	0	2.8	2.3	0	7.7	0	

The current demonstration experiment and results are clearly very preliminary, but they demonstrate the feasibility of using the cluster imaging system to scan nine or even more participants simultaneously in a realistic, face-to-face social interaction environment. The MBN showed that, during the drumming, all the participants' brains were linked by either direct or indirect paths, providing possible routes through which cooperation information may transfer. The topological indices indicate different nodes have distinct degree and global/local efficiency, suggesting diversity of roles during the group cooperation task. Although the neural basis of this MBN still awaits further study, our platform provides a new avenue by which to explore the intricate neural inter-communication process. In future studies, we can extend the present group drumming experiment with more experimental conditions and larger sample sizes. The drumming sound data can also be leveraged in the analysis to investigate the MBN's behavioral significance.

## Discussion

Social interactions have critical significance in all aspects of human life, from individuals' development to the species' evolution. With the goal of studying the brain mechanisms behind social interactions, social neuroscientists have always sought methods that can measure and analyze the brains of interacting people in more realistic social crowds and more natural social environments (Hari and Kujala, 2009; Liu and Pelowski, 2014). In the present study, we propose CIMBN as a research framework for scanning and modeling a cluster of interacting brains. We extend multi-brain hyperscanning techniques from both imaging and data analysis aspects and provide a new tool for studying the neural correlates of social interactions among a crowd of people.

Our work affords a way to greatly extend parallel measurement capacity, which is an important technical index of hyperscanning techniques. Larger simultaneous measurement capacity is very important for the advancement of social neuroscience. Humanity has always been organized in social groups of various scales, from the nuclear family to the nation-state, which raises a variety of interesting and valuable scientific questions for social neuroscience, such as the nature of cooperation and competition (Bowles, 2006; Vollan and Ostrom, 2010), collective intelligence (Woolley et al., 2010), group cohesion and conflict (Pelled, 1996; Pescosolido and Saavedra, 2012), social facilitation and loafing (Zajonc, 1965; Ying et al., 2014), group structure (Palla et al., 2007), leadership (Beaman et al., 2012), and group-personality (Kramer et al., 2014). Most of these social group behaviors usually occur with the participation of a crowd of people, which makes it important to track all interacting brains simultaneously. In the present work, we established a cluster-imaging technique for simultaneously scanning multiple brains and demonstrate a measurement capacity of nine participants. To the best of our knowledge, this is by far the highest capacity hyperscanning system, and it can be further extended to accommodate dozens or even hundreds of participants by simply integrating more fNIRS systems, with negligible ecological validity loss.

Another contribution of our work is a new approach for modeling and analyzing such cluster-imaging data. Existing analysis methods for hyperscanning studies involving more than two participants mainly work on two levels. One is the single-brain level, which treats all the interacting brains separately (Babiloni et al., 2011, 2012; Tomlin et al., 2013; Smith et al., 2014), and another is the paired-brain level, which focuses on the hyperlinks (i.e., the cross-brain connectivities) between two brains (Babiloni et al., 2006, 2007b). These analysis methods showed potential in pioneering studies. However, when the number of simultaneously scanned brains greatly increases, the social interaction processes which can be analyzed will be much more complex. People combine to form small groups through interpersonal interactions and small groups combine to form larger groups through intergroup interactions. Usually, the large groups' characteristics are far different from that of the small groups. That is to say, the effects of group social interactions cannot be simply understood as “one plus one makes two.” This non-linearity property of social interactions suggests that, for a larger social group made up of many people, existing analysis methods may not be enough to thoroughly study the neural underpinnings of group social behaviors, even by assembling all single-brain and paired-brain information. Therefore, we propose a new approach—MBN analysis—for modeling and analyzing the cluster imaging data. Under the framework of CIMBN, the neural correlates of a group's social behaviors can be investigated at different scales, such as individuals, subgroups, and the whole group, using the MBN's nodal properties, sub-network properties, and whole-network properties. Additionally, the changes of the MBN during social interactions can also be studied. These dynamics, including evolutionary events such as growth, merging, contraction, and splitting (Palla et al., 2007), may provide new insights into the mechanisms for the propagation of behavior, knowledge, and emotion in social groups.

## Limitations and future work

As a general framework for multi-brain imaging and modeling, CIMBN still has some limitations and may benefit from further development. In terms of the clustering imaging technique, the optical fibers of fNIRS systems sometimes limit the freedom of the social interactions and bring difficulties in cable arrangement, especially when the number of fNIRS systems increases. In the future, wireless fNIRS systems may be employed to address this problem.

EEG, a frequently-used, low-cost, and non-invasive brain imaging modality, can be added into the CIMBN framework to complement fNIRS for cluster imaging. In contrast with fMRI and fNIRS, which record cerebral hemodynamic changes, EEG records the brain's neuroelectrical activity and has very high temporal resolution. If fNIRS and EEG can be combined to realize concurrent recording for cluster imaging, it will add flexibility in terms of the types of analyses we can conduct and thus assist the investigation of the neural underpinnings of group social interactions.

CIMBN can also be extended to be internet-compatible, allowing simultaneous recording at different geographical locations with synchronization and data exchange occurring via the internet. This would not only increase imaging capacity by combining fNIRS systems from different laboratories, but also enable researchers to break through geographical constraints to investigate long-distance social interaction processes, such as social exchanges between cultures and social interactions in cyberspace.

Although promising, the present MBN analysis method is still preliminary and exploratory. Work is needed to further develop the method. In future work, we plan to clarify the impact of factors influencing MBN analysis, such as the noise characteristics of cluster imaging data and different definitions for network nodes (e.g., single ROI or multiple ROIs) and edges (e.g., threshold selection for edge retention). In addition, we plan to conduct social group interaction experiments with multiple sessions to validate the test-retest reliability of the MBN method. Finally, we plan to apply the MBN analysis method to a diverse series of group social interaction experiments for further validation and improvement.

## Statement of rights and ethics

All participants provided written informed consent for the experiment, and they also agreed to the publication of their photographs. The experimental protocol was approved by the Institutional Review Board at the State Key Laboratory of Cognitive Neuroscience and Learning, Beijing Normal University.

### Conflict of interest statement

The authors declare that the research was conducted in the absence of any commercial or financial relationships that could be construed as a potential conflict of interest.
